# Defending and defining compulsive behaviour in addiction

**DOI:** 10.1111/adb.13427

**Published:** 2024-08-12

**Authors:** Karen D. Ersche

**Affiliations:** ^1^ Department of Psychiatry University of Cambridge Cambridge UK; ^2^ Department of Addictive Behaviour and Addiction Medicine, Central Institute of Mental Health University of Heidelberg Mannheim Germany

**Keywords:** addiction, compulsivity, habit, habit theory

Heinz and colleagues provide interesting insights into the clinical presentation of drug‐taking habits and the associated difficulties of breaking them. Although they do not question the existence of habits in addiction, they raise concerns about the psychological construct of habit and its role in the development of compulsivity in addiction.

The authors doubt that compulsive behaviour in addiction arises from a predominance of habits over goal‐directed behaviour, as suggested by the habit theory.^1^ Their arguments are based on descriptions of differences in clinical phenotypes of compulsivity in addicted patients and patients with obsessive‐compulsive disorder (OCD), without mentioning the many commonalities. For example, they explain that avoidance behaviour in OCD patients is negatively reinforced through the relief of anxiety but do not say that hoarding behaviour in OCD patients is positively reinforced.^2^ Likewise, they emphasise that the use of alcohol is positively reinforced by its pleasurable effects but do not mention the fact that negative reinforcement underlies chronic opioid use.^3^ Similar commonalities are also evident in the brain, as reflected by an overlapping neuropathology underlying self‐reported compulsivity in both OCD and addiction.^4^ Focussing solely on different manifestations of orbitofrontal dysfunction (i.e., cases of overactivity or underactivity) distracts from the fact that the same system is impaired in both disorders but expressed in different ways. I wonder whether the authors' questioning of the role of compulsivity in addiction derives from an understanding that equates the psychological concept of compulsivity (i.e., the maladaptive continuation/perseveration of behaviour) with clinical symptoms of compulsions. Compulsive symptoms can of course be expressed in many different ways, as exemplified by the authors' clinical case of an OCD patient with comorbid compulsive alcohol use and gambling behaviour. Whilst there are variations of compulsive symptoms across different disorders, the psychological concept underpinning these behavioural manifestations is the same, namely, a reflection of ongoing actions that have become inappropriate to the immediate context.

Moreover, it is worth clarifying that the habit theory does not contradict their observations. Habits (including habitual drug use) do not necessarily develop into compulsions because most people are able to break their habits. If habits are, however, learned under the influence of drugs or stress, the formation of habits is facilitated. In people with impaired prefrontal inhibitory control (such as patients with OCD or addiction), habits run the risk of persisting even if they no longer produce the desirable effects or lead to adverse consequences. This only affects a minority of drug users, as just 15%–20% are thought to develop addiction.^5^ Importantly, those who make this transition to addiction do not turn into ‘autonomous robots’ but are prone to continue using drugs in response to drug‐associated cues despite good intentions to stop using and full awareness of the severe adverse consequences, perpetuated by persistent drug use. Heinz and colleagues rightly point out that negative consequences in humans are not imminent (in contrast to preclinical studies), but immediacy is not necessary. Knowledge about the negative outcome is sufficient. Indeed, most addicted patients are undeterred by knowing the harm, perpetuated by continued drug use.

Possibly, the authors' criticism of compulsivity derives from their understanding of the habit construct, which they feel needs to be described in ‘dimensional’ terms. Unfortunately, they do not explain what they mean by ‘dimensional habits’. It may be that they refer to computational models, which explain behaviour through mathematical algorithms, thus providing a continuous measure of habit strength rather than defining it by the absence of goal‐directed actions. Yet many computational models still require empirical validation,^6^ as exemplified by the poor construct validity of habits assessed through algorithms of model‐free learning.^7^, ^8^ Alternatively, Heinz and colleagues may refer to the notion that a construct, which supposedly defines addiction, should correlate with measures of addiction severity. Although habitual drug use does not equate with addiction, several studies have shown relationships between habit strength and indicators of addiction severity.^9^, ^10^, ^11^, ^12^ However, the authors solely focus on criticism that is based on qualitative comparisons^13^ of studies but not on quantitative meta‐analyses.

Importantly, the authors' understanding of habit differs conspicuously from that of behavioural neuroscience, which describes behaviour as a process of distinct associative learning mechanisms, each dependent on defined corticostriatal loops. The initial learning phase, when the behaviour and its consequences are learned in a stable context, relies on the ventral striatum, adjunct limbic structures and the medial prefrontal cortex. Once the behaviour is established and the consequences are predictable, deliberation is no longer needed, and behaviour can be executed automatically. Consequently, sensorimotor regions in the dorsal striatum and connected sensory and motor cortices take over control. As learning is a process, loops subserving behaviour may well overlap, but only one of the two systems can be in control. In cases of ambiguity, the model suggests an arbitration system of executive function that allocates control to either the goal‐directed or the habit system.^14^ These systems are then tested in an experimental setting by using manipulations that either make the consequences of the learned action meaningless (i.e., outcome devaluation) or break the link between the action and the outcome (i.e., contingency degradation). If the behaviour continues regardless, it is assumed that it has become habitual, but if the behaviour discontinues (because it no longer makes sense), then it is under goal‐directed control. Importantly, the theory does not say that drug use must always be habitual, nor does it question patients' ability reflect on their behaviour. On the contrary, the theory predicts that patients are more susceptible to cues but struggle to control their response due to weakened prefrontal inhibition.

The question that Heinz and colleagues raise is, however, a valid one, namely, whether habits provide the *only* route to compulsions. Possibly not, impaired inhibitory control does not only affect the regulation of habitual behaviour but also goal‐directed actions. From a behavioural economic perspective, compulsive drug‐seeking derives from decisional choices (goals), which have narrowed towards drugs over the course of the disorder.^15^ Drug‐related rewards are then attributed higher subjective value than alternative rewards. So, when unpredictable opportunities for drug use occur, the excessive motivation for the drug and the low attractiveness of non‐drug‐related options may cause an increase in drug‐related activities. Evidently, drugs are not always used in the same stable environment, but if they are, drug‐taking may become habitual. On unpredictable occasions, however, drug‐taking may remain goal‐directed. In both scenarios, drug taking can spiral out‐of‐control in vulnerable individuals.

Although the self‐destructive pattern of compulsive drug‐seeking probably reflects most peoples' understanding of addictive behaviour, there is disagreement within the psychiatric community about the role of compulsions in addiction. This may be due, at least in part, to the different diagnostic criteria used in the *Diagnostic and Statistical Manual of Mental Disorders, Fifth Edition* (DSM‐5) and the *International Classification of Diseases, Eleventh Edition* (ICD‐11). Although both manuals stipulate the lack of control over drug use as the hallmark of addiction, only the DSM‐5 lists compulsive patterns of drug use as an additional presentation of impaired control, namely, the persistent use of drugs despite problems and/or knowledge of harm caused or exacerbated by further drug use (which is an obvious definition of compulsive drug‐seeking behaviour). Presumably Heinz and colleagues are adopting the ICD‐11 criteria, which do not include compulsive behaviour. However, this diagnostic discrepancy requires urgent resolution. Understanding the different mechanisms by which patients lose control over their drug use is pivotal for addressing their needs adequately in treatment.

Inasmuch as the authors' insights on the diversity of clinical phenotypes provide interesting anecdotes, they are difficult to reconcile with the existing neurobehavioural basis of addiction. Rejecting the habit theory of addiction might be desirable scientifically, but this requires a superior theory to replace it, and it is unclear what Heinz and colleagues have in mind. Given that there is much evidence for goal‐directed and habit neural systems controlling motivation and behaviour, an obvious step forward would be to test the habit theory in human addiction, as many neuroscientists have already done. Neuroscientific research also provides a promising pathway in elucidating the aetiology of addiction, despite the difficulties in translating experimental paradigms from animals to humans due to legal and ethical constraints. Of course, animal models could be improved further to narrow the gap between the laboratory and complex human environments. Yet the translation to humans remains essential, and the development of theoretically sophisticated human experimental paradigms that can be delivered in ecologically valid environments will thus be key for the future of human addiction research.
